# Hippocampal Place Cell Sequences Are Impaired in a Rat Model of Fragile X Syndrome

**DOI:** 10.1523/JNEUROSCI.1978-24.2025

**Published:** 2025-03-03

**Authors:** Margaret M. Donahue, Emma Robson, Laura Lee Colgin

**Affiliations:** ^1^Center for Learning and Memory, The University of Texas at Austin, Austin, Texas 78712; ^2^Institute for Neuroscience, The University of Texas at Austin, Austin, Texas 78712; ^3^Department of Neuroscience, The University of Texas at Austin, Austin, Texas 78712

**Keywords:** Fragile X syndrome, hippocampus, place cells, replay, sharp wave-ripple, theta

## Abstract

Fragile X syndrome (FXS) is a neurodevelopmental disorder that can cause impairments in spatial cognition and memory. The hippocampus is thought to support spatial cognition through the activity of place cells, neurons with spatial receptive fields. Coordinated firing of place cell populations is organized by different oscillatory patterns in the hippocampus during specific behavioral states. Theta rhythms organize place cell populations during awake exploration. Sharp wave-ripples organize place cell population reactivation during waking rest. Here, we examined the coordination of CA1 place cell populations during active behavior and subsequent rest in a rat model of FXS (*Fmr1* knock-out rats). While the organization of individual place cells by the theta rhythm was normal, the coordinated activation of sequences of place cells during individual theta cycles was impaired in *Fmr1* knock-out rats. Furthermore, the subsequent replay of place cell sequences was impaired during waking rest following active exploration. Together, these results expand our understanding of how genetic modifications that model those observed in FXS affect hippocampal physiology and suggest a potential mechanism underlying impaired spatial cognition in FXS.

## Significance Statement

Fragile X syndrome (FXS) is a neurodevelopmental disorder that can cause impaired memory and atypical spatial behaviors such as “elopement” (i.e., wandering off and becoming lost). Activity in the CA1 subregion of the hippocampus supports spatial memory and spatial cognition, making it an important candidate to study in the context of FXS; however, how neuronal population activity in CA1 is affected by FXS is poorly understood. In this study, we found that the coordination of populations of CA1 neurons during active behavior and waking rest was impaired in a rat model of FXS. These results reveal hippocampal physiological deficits that may contribute to cognitive impairments in FXS.

## Introduction

Fragile X syndrome (FXS) is a widespread neurodevelopmental disorder that is caused by epigenetic suppression of the X chromosome-linked *Fmr1* gene and subsequent loss of fragile X messenger ribonucleoprotein (FMRP) (Kremer et al., 1991; Pieretti et al., 1991; De Boulle et al., 1993; Mikiko et al., 1995). Patients with FXS are impaired in memory tasks (Jäkälä et al., 1997; Cornish et al., 1998; Gallagher and Hallahan, 2012) and often display elopement behaviors (Machalicek et al., 2014; Muller et al., 2019). Mouse and rat models of FXS (“FXS mice” or “FXS rats,” respectively) have been produced by knocking out the *Fmr1* gene (Bakker et al., 1994; Tian et al., 2017). Spatial memory deficits have been reported for both FXS mice and FXS rats (Bakker et al., 1994; Van Dam et al., 2000; Mineur et al., 2002; Till et al., 2015; Tian et al., 2017; Asiminas et al., 2019). FMRP is an RNA-binding protein (Siomi et al., 1993) that interacts with many neuronal proteins (Darnell et al., 2011; for a review, see Darnell and Klann, 2013) and is highly expressed in the hippocampus (Ludwig et al., 2014).

The hippocampus is thought to support memory and spatial cognition through the activity of place cells, neurons with spatial receptive fields known as “place fields” (O’Keefe and Dostrovsky, 1971; O’Keefe, 1976). Populations of place cells form sequences that represent trajectories through an environment. These sequential firing patterns are coordinated by hippocampal rhythms that are differentially associated with behavioral states. During active exploration of an environment, place cells are coordinated by the theta rhythm, a ∼6–10 Hz oscillation occurring prominently in local field potential (LFP) recordings from the hippocampus (Buzsáki, 2002). As an animal moves through a cell's place field, place cells fire at progressively earlier phases of the theta rhythm in a phenomenon known as “theta phase precession” (O’Keefe and Recce, 1993). Coordinated theta phase precession across multiple place cells with adjacent place fields would be expected to result in an organized sequence of spikes within an individual theta cycle that represent a rat's previous, current, and future locations. Such sequential activation of place cells within a single theta cycle has been observed in rats and termed a “theta sequence” (Foster and Wilson, 2007). Previous work has suggested that hippocampal networks are hypersynchronized in FXS (Talbot et al., 2018; Arbab et al., 2018a), which could impair organization of cells in theta sequences. However, the activity of theta-coordinated sequences of place cells is yet to be investigated in a rodent model of FXS.

Sequences of place cells that were activated during active exploration of an environment are reactivated or “replayed” in a time-compressed manner during subsequent waking rest and non-REM sleep (Kudrimoti et al., 1999; Lee and Wilson, 2002). Place cell replay co-occurs with characteristic events in the hippocampal LFP called sharp wave-ripples (Kudrimoti et al., 1999; Nádasdy et al., 1999; Lee and Wilson, 2002). Sharp wave-ripples during sleep are abnormal in FXS mice (Boone et al., 2018), suggesting that coordination of place cell populations during sleep and rest may be disrupted in FXS. However, replay of place cell sequences has not been examined in FXS models.

Here, we examined the activity of coordinated sequences of CA1 place cells in FXS rats traversing a familiar circular track and subsequently resting. During active behaviors, we found that the coordination of individual place cells by the theta oscillation was normal in FXS rats. However, theta-coordinated sequences of place cells in FXS rats were less temporally compressed and represented shorter paths than those in wild-type (WT) control rats. Furthermore, replay of place cell sequences during rest was abnormally slow in FXS rats, with replay events exhibiting longer durations and representing less temporally compressed spatial trajectories than in WT rats. These findings raise the possibility that the abnormal coordination of sequences of hippocampal place cells may contribute to impairments in spatial memory and cognition in FXS.

## Materials and Methods

Additional data collected from one of the rats (rat 418) used in this study were presented in a previous study (Robson et al., 2025). Surgery, data acquisition, histology, and spike sorting methods in this paper are identical to methods described in that study and restated below.

### Subjects

Twelve male Sprague Dawley rats (Inotiv) were used for this study. Six rats were *Fmr1* knock-out rats (*SD-Fmr1-nulltm1Sage*), and six were littermate WT control rats. As FXS is an X chromosome-linked disorder, FXS has a higher prevalence and increased symptom severity in males. Thus, we chose to use male rats for this study. Rats were between the ages of 3 and 11 month old at the time of surgery. Prior to surgery, rats were double- or triple-housed in genotype-matched groups. After surgery, rats were singly housed in custom-built acrylic cages (40 × 40 × 40 cm) containing enrichment material (wooden blocks, paper towel rolls, etc.) and maintained on a reverse light cycle (light, 8 P.M.–8 A.M.). Rats were housed next to their former cage mates after recovering from surgery and throughout behavioral testing. Rats recovered from surgery for at least 1 week before behavioral training resumed. All behavioral experiments were performed during the dark cycle. When necessary to encourage spatial exploration, one rat (rat 316) was placed on a food-deprivation regimen. While on the regimen, this rat maintained ∼98% of his free-feeding body weight. Following the completion of all recording experiments, a small piece of ear tissue was collected from each rat to verify genotype. All experiments were conducted according to the guidelines of the United States National Institutes of Health Guide for the Care and Use of Laboratory Animals and under a protocol approved by the University of Texas at Austin Institutional Animal Care and Use Committee.

### Surgery and tetrode positioning

“Hyperdrives” with 14 independently movable tetrodes were implanted in eight of the rats. Hyperdrives with 21 independently movable tetrodes were implanted in four of the rats. Implants were positioned above the right dorsal hippocampus (anterior–posterior −3.8 mm from the bregma, medial–lateral −3.0 mm from the bregma). To implant and stabilize the hyperdrives, 11 bone screws were affixed to the skull, and the base of the implant and the screws were covered in dental acrylic. Two of the screws were connected to the recording drive ground. Prior to surgery, tetrodes were built from 17 μm polyimide-coated platinum–iridium (90/10%) wire (California Fine Wire). The tips of tetrodes designated for single-unit recording were plated with platinum to reduce single-channel impedances to ∼150–300 kOhms. All tetrodes were lowered ∼1 mm on the day of surgery. Thereafter, tetrodes were slowly lowered to the hippocampal pyramidal cell body layer over the course of several weeks except for one tetrode that was designated for use as a reference for differential recording. This reference tetrode was placed in an electrically quiet area ∼1 mm above the hippocampus and adjusted as needed to ensure quiescence. All four wires of this tetrode were connected to a single channel on the electrode interface board. The reference signal was duplicated using an MDR-50 breakout board (NeuraLynx) and recorded continuously to ensure that unit activity or volume conducted signals of interest were not detected. Another tetrode was placed in the apical dendritic layer of CA1 to monitor LFPs and guide placement of the other tetrodes using estimated depth and electrophysiological hallmarks of the hippocampus (e.g., sharp wave-ripples).

### Data acquisition

Data were acquired using a Digital Lynx system and Cheetah recording software (NeuraLynx). The recording setup has been described in detail previously (Hsiao et al., 2016; Zheng et al., 2016). Briefly, LFP signals from one randomly chosen channel per tetrode were continuously recorded at a 2,000 Hz sampling rate and filtered in the 0.1–500 Hz band. Input amplitude ranges were adjusted before each recording session to maximize resolution without signal saturation. Input ranges for LFPs generally fell within ±2,000 to ±3,000 μV. To detect unit activity, all four channels within each tetrode were bandpass filtered from 600 to 6,000 Hz. Spikes were detected when the filtered continuous signal on one or more of the channels exceeded a threshold of 55 µV. Detected events were acquired with a 32,000 Hz sampling rate for 1 ms. For both LFPs and unit activity, signals were recorded differentially against a dedicated reference channel (see above, Surgery and tetrode positioning).

Videos of rats’ behavior were recorded through the NeuraLynx system with a resolution of 720 × 480 pixels and a frame rate of 29.97 frames/s. Rat position and head direction were tracked via an array of red and green or red and blue light-emitting diodes (LEDs) on a HS-54 or HS-27 headstage (NeuraLynx), respectively.

### Behavior

Rats were trained to run unidirectionally on a 1-m-diameter circular track. The track was 0.5 m in height. Rats ran four 10 min sessions on the track per day. Intersession rest periods were 10 min, and the rat rested in a towel-lined flowerpot in the recording room during rest sessions. Rats additionally rested in the pot for 10 min prior to the start of the first track running session and after the final session for a total of five rest sessions per day. Small pieces of sweetened cereal or cookies were placed at one or two locations on the circular track to encourage running. The reward locations were kept consistent within each day but changed daily in order to prevent accumulation of place fields at the reward site (Hollup et al., 2001). To ensure that rats were familiarized with the environment prior to recording, rats completed a minimum of 2 d of the full recording session (i.e., all five rest sessions and all four run sessions) before data collection began. To compare behavior between genotypes, we calculated the run speed on the circular track and the number of laps completed per session. To ensure exclusion of pauses in running behavior and periods of immobility, we included only times when the rat was traveling at speeds >5 cm/s when calculating the run speed.

### Histology and tetrode localization

Following recording, rats were perfused with 4% paraformaldehyde solution in phosphate-buffered saline. Brains were cut coronally in 30 μm sections using a cryostat. Brain slices were immunostained for the CA2 marker Purkinje cell protein 4 (PCP4), allowing differentiation of all subregions of the hippocampus (CA1, CA2, and CA3). Sections were initially washed and blocked in 10% normal goat serum in TBS. Sections were incubated overnight with rabbit anti-PCP4 (1:200, Sigma-Aldrich catalog #HPA005792) diluted in TBS containing 0.05% Tween. The next day, sections were washed and incubated overnight with a secondary fluorescent antibody (Alexa Flour 555 anti-rabbit, Thermo Fisher Scientific). All washes and incubations were performed at room temperature. Slides were mounted using DAPI Fluoromount-G (Thermo Fisher Scientific). Tetrode recording sites were identified by comparing locations across adjacent sections.

### Spike sorting

Spike sorting was performed manually using a graphical cluster-cutting software (MClust, A.D. Redish, University of Minnesota) run in MATLAB (MathWorks). Spikes were sorted using two-dimensional representations of waveform properties (i.e., energy, peak, and peak-to-valley difference) from four channels. A single unit was accepted for further analysis if the associated cluster was well isolated from, and did not share spikes with, other clusters on the same tetrode. Units were also required to have a minimum 1 ms refractory period. Units with mean firing rates above 5 Hz were considered putative interneurons and excluded from further analyses. To be included in the replay event firing analyses, a unit was required to have valid clusters in both the active exploration and rest sessions. Place cell yields for each rat for the active behavior and replay event analyses are reported in Tables 1 and 2, respectively.

**Table 1.** Total number of place cells recorded from each rat during active behavior

Genotype	Rat	Number of place cells
WT	326 (“Zachariah”)	94
WT	334 (“Chickpea”)	4
WT	335 (“Couscous”)	5
WT	392 (“Danish”)	29
WT	416 (“Desmond”)	60
WT	418 (“Hugo”)	71
FXS	316 (“Yuki”)	21
FXS	330 (“Aries”)	99
FXS	394 (“Mr. Eko”)	3
FXS	395 (“Elijah”)	36
FXS	445 (“Paddy”)	20
FXS	442 (“Pippin”)	69

**Table 2.** Total number of place cells recorded from each rat during replay events

Genotype	Rat	Number of place cells
WT	326 (“Zachariah”)	93
WT	392 (“Danish”)	16
WT	416 (“Desmond”)	60
WT	418 (“Hugo”)	71
FXS	316 (“Yuki”)	21
FXS	330 (“Aries”)	98
FXS	395 (“Elijah”)	18
FXS	442 (“Pippin”)	69

### Place cell analyses

**Figure 1.**
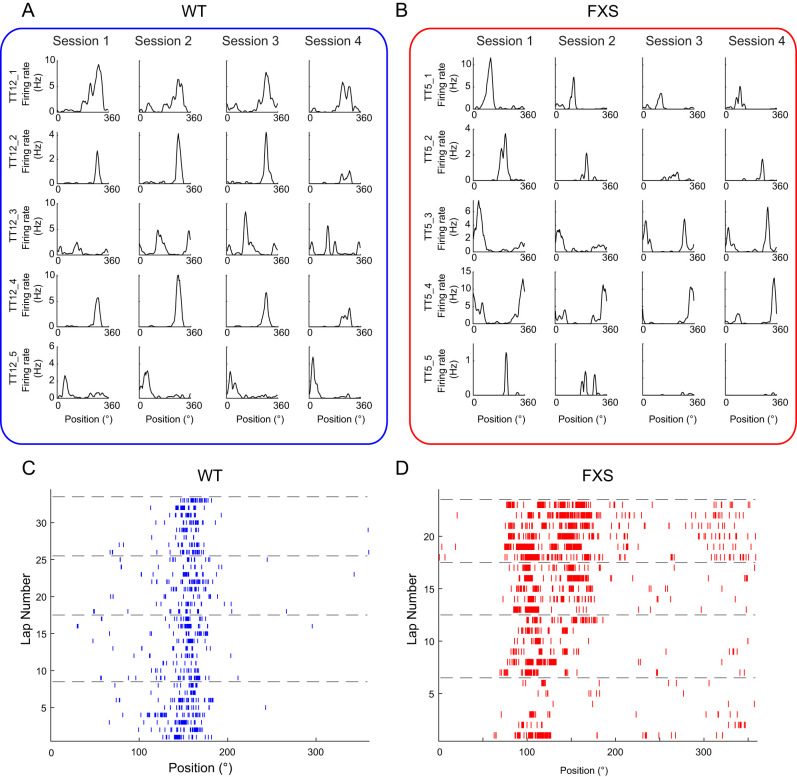
Example CA1 place cells across four recording sessions from WT and FXS rats. ***A***, ***B***, Firing rates across positions on the circular track are shown for all place cells (rows) recorded from one example tetrode across all four sessions (columns) in an example day from a WT (***A***) and a FXS (***B***) rat. ***C***, ***D***, Example spike raster plots for successive laps within a day for a WT (***C***) and a FXS (***D***) place cell. Dashed lines separate different sessions.

Only cells from tetrodes in CA1 were included in analyses (see above, Histology and tetrode localization). Firing rate maps were created for each single unit using methods based on those used in our prior studies (Hwaun and Colgin, 2019; Zheng et al., 2021; see Fig. 1*A*,*B* for example rate maps). The circular track was divided into 4° bins. The number of spikes fired by a unit was divided by the time spent in each bin. Spikes that occurred at times when a rat was moving at speeds <5 cm/s were excluded. This rate map was then smoothed with a Gaussian kernel (standard deviation, 8°). Rate maps were calculated individually for each run session and for all run sessions in each day. To be included for further analysis, the day-averaged rate map of a cell had to reach a minimum peak firing rate of 1 Hz.

**Figure 2.**
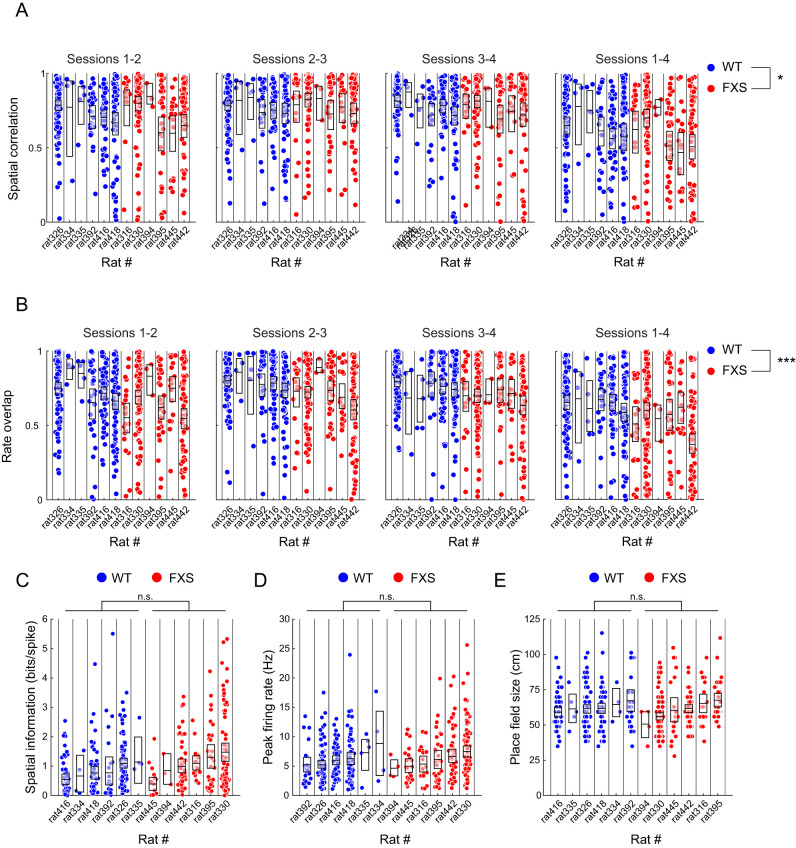
Place cell firing rate maps in FXS rats were unstable across sessions but otherwise exhibit normal place field properties. ***A***, Spatial correlation coefficients are shown across sessions pairs for each rat. Spatial correlation values were lower in FXS rats than WT rats, indicating impaired stability of place cell responses in FXS rats. ***B***, Rate overlap values are shown across session pairs for each rat. Rate overlap values were lower in FXS rats than in WT rats, indicating highly variable firing rates of place cells across sessions in FXS rats. ***C***, There was no difference in spatial information of place cells between WT and FXS rats. ***D***, Peak firing rates of place cells did not differ between WT and FXS rats. ***E***, The place field size did not differ between WT and FXS rats. For all plots, each dot represents a measure from one place cell. Boxes represent 95% confidence intervals of the mean for each rat.

To examine stability of place cell rate maps, a Pearson's correlation coefficient *R* (“spatial correlation”) was calculated for each unit between pairs of rate maps from different sessions (Fig. 2*A*). We additionally calculated the rate overlap between pairs of rate maps from different sessions for each cell to determine whether a cell's firing rate changed significantly across sessions (Fig. 2*B*). The rate overlap was calculated by taking the ratio of mean firing rates between two sessions, with the larger firing rate as the denominator (Colgin et al., 2010).

Spatial information was calculated using the following formula (Skaggs et al., 1996):

$$\mathrm{Spatial}\;\mathrm{information}=\;\sum P_{i}\frac{\lambda_{i}}{\lambda }\log2\frac{\lambda_{i}}{\lambda },$$

where *i* is an index of spatial bins, *P_i_* is the probability of a rat being in the *i*th bin, *λ_i_* is the mean firing rate in the *i*th bin, and *λ* is the overall mean firing rate of the cell (Fig. 2*C*).

To identify place fields (Fig. 2*D*,*E*), we first *z*-scored the firing rates across locations in the rate map. Potential place fields were identified as locations where the *z*-scored firing rate was ≥2. Identified place fields were bounded by locations where *z*-scored firing rates fell below 0.5. To be included for further analysis, the peak firing rate in a place field had to be at least 1 Hz, and the minimum length of a place field had to be at least 18° (∼15 cm).

### Phase precession analysis

**Figure 3.**
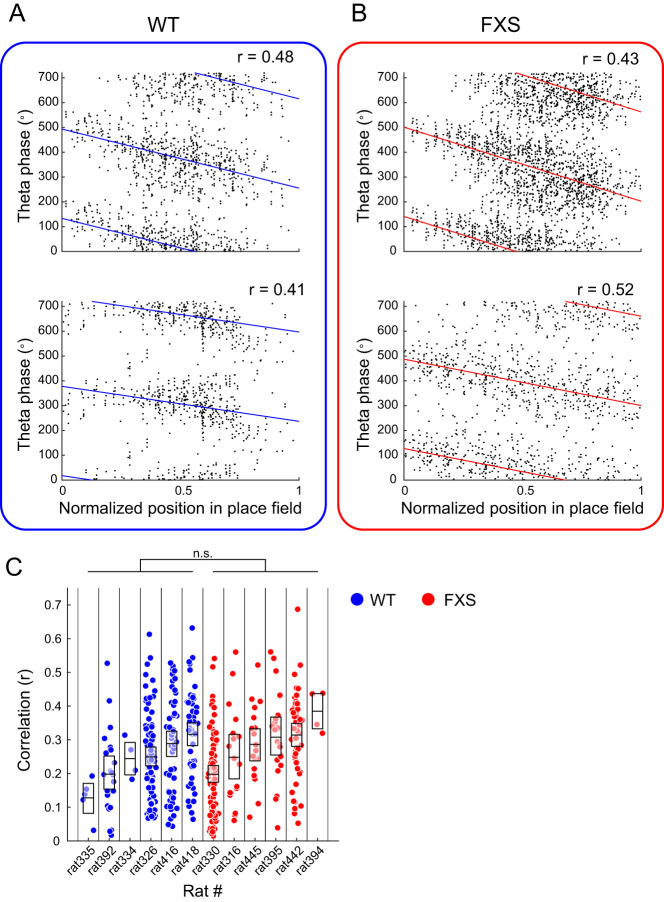
Theta phase precession was preserved in FXS rats. ***A***, ***B***, Place cell phase precession plots are shown for two example place cells from (***A***) WT and (***B***) FXS rats. Each dot represents the theta phase associated with each spike and corresponding normalized position in the cell's place field. The solid line represents the correlation between the theta phase and normalized position in place field. The magnitude of the correlation (*r*) is shown (top right) for each cell. ***C***, The correlation between theta phase and normalized position in a place field did not differ for place cells from WT and FXS rats. Each dot represents the correlation measure for one cell. Boxes represent 95% confidence intervals of the mean for each rat.

Phase precession analysis was performed similarly to the analysis described in our previously published work (Bieri et al., 2014). The LFP signal from every tetrode that recorded CA1 cells identified as place cells was bandpass filtered in the theta range (i.e., between 6 and 10 Hz). The phase of the theta oscillation was then estimated using a Hilbert transform. Locations within each place field were normalized between 0 and 1. For each spike that a cell fired within its place field on all sessions within a day, the theta phase at the spike time was estimated using the transformed signal from the tetrode on which it was recorded. The normalized distance through the place field at the spike time was also determined. Spikes that occurred while the rat was traveling at a speed of <5 cm/s were excluded. A place cell had to fire a minimum of 50 spikes within its place field in order to be included in theta phase precession analysis. Circular–linear regression was then performed with theta phase as the circular variable and normalized distance through the place field as the linear variable (Fig. 3*A*,*B*). The correlation coefficient for the relationship between theta phase and normalized distance (Fig. 3*C*) was calculated using the *circ_corrcl* function from the Circular Statistics toolbox in MATLAB (Berens, 2009; https://www.mathworks.com/matlabcentral/fileexchange/10676-circular-statistics-toolbox-directional-statistics).

### Bayesian decoding analysis

We used a Bayesian decoding algorithm (Zhang et al., 1998) to estimate posterior probability distributions of angular positions represented by the spiking activity of CA1 place cell populations during track running. The probability of a rat being at position *x* given the number of spikes *n* that occurred within a given time window was defined using Bayes’ rule as follows:

$$P(x|n)=\;\frac{P(n|x)\;*\;P(x)}{P(n)},$$

where *P(n | x)* was estimated using the averaged position tuning of each unit across all four sessions. The probability of a rat being at any given position on the circular track *P(x)* was set to 1 to avoid biasing the decoder toward any particular position on the circular track. The normalizing constant *P(n)* was set such that the posterior probability distribution *P(x | n)* summed to 1.

### Decoding accuracy analysis

**Figure 4.**
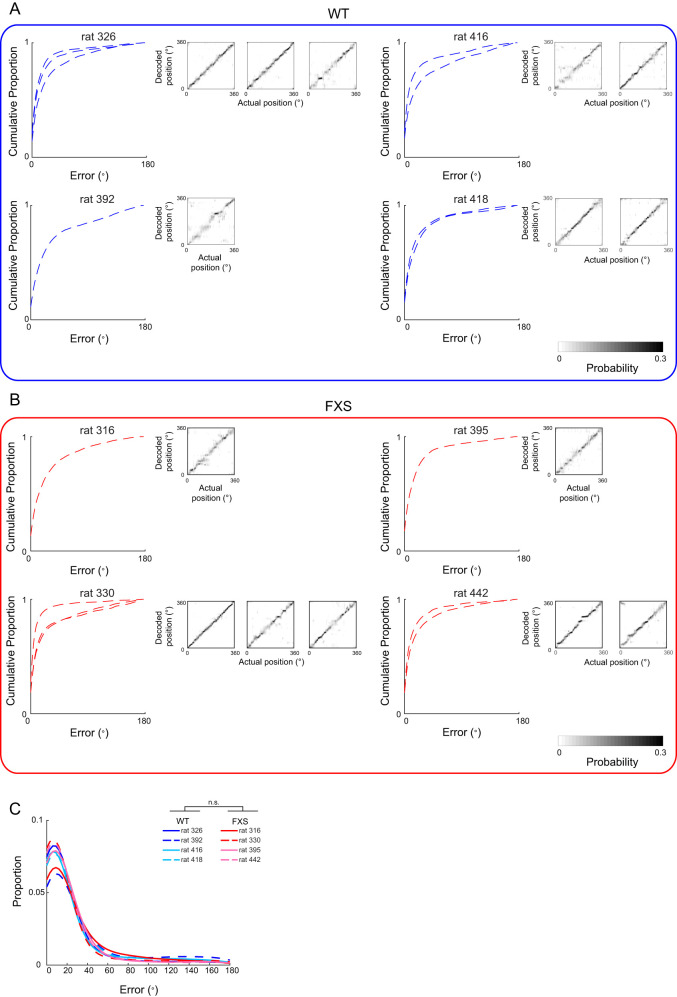
Decoding accuracy in WT and FXS rats. ***A***, ***B***, Cumulative decoding error and confusion matrices are shown for decoded place cell populations from WT (***A***) and FXS (***B***) rats. Individual lines on cumulative error plots represent individual days. Insets show confusion matrices from each day for each rat. Only days that met the decoding criteria are shown. ***C***, There was no difference in decoding error values between WT and FXS rats. Each line represents the distribution of decoding error values from one rat.

To verify that a given day's place cell yields were sufficient to accurately decode a rat's positions on the circular track, we used a decoding accuracy analysis similar to the methods described in our previously published work (Hwaun and Colgin, 2019; Zheng et al., 2021). Positions were decoded for all times when a rat was traveling at speeds over 5 cm/s in 500 ms windows with 100 ms steps. To create confusion matrices (Fig. 4*A*,*B*), we calculated the average decoded probability for all times when a rat was at a given position. To determine the decoding error, we defined the decoded position as the position with maximal decoded probability for each time bin. The decoding error was then defined as the difference between a rat's actual position and the corresponding decoded position. The cumulative error distributions for each day were then determined (Fig. 4*A*,*B*). For a day of recordings to be included in theta sequence event and replay event analysis, its cumulative error distribution had to reach 50% at error values <20° (Davidson et al., 2009; Hwaun and Colgin, 2019). Days that reached this criterion are shown in Figure 4, *A* and *B*. The overall error distributions for rats with days that met this criterion are shown in Figure 4*C*.

### Detection and analysis of theta sequences

**Figure 5.**
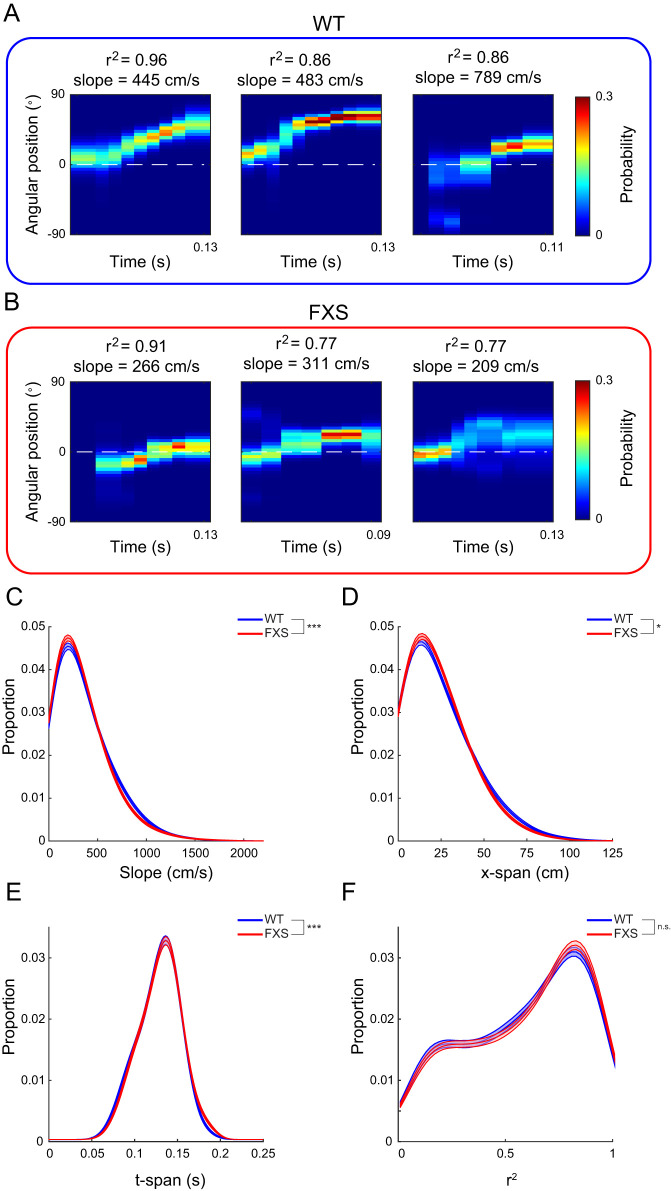
Theta sequence events coded paths that were less temporally compressed and shorter in FXS rats than in WT rats. ***A***, ***B***, Example theta sequence events are shown for WT (***A***) and FXS (***B***) rats. Position (on the *y*-axis) is shown relative to the mean of the rat's actual position during the theta sequence event (indicated by white dashed line). The associated *r*^2^ and slope values are shown for each event. ***C***, Theta sequences’ slopes were lower in FXS rats than WT rats, indicating that theta sequences were less temporally compressed in FXS rats. ***D***, The *x*-span values of theta sequences were lower in FXS rats than in WT rats, indicating that theta sequences represented relatively short spatial paths in FXS rats. ***E***, The duration (*t*-span) of theta sequences was higher in FXS rats than in WT rats. ***F***, There was no difference in *r*^2^ values between FXS and WT rats, indicating that theta sequences represented coherent paths through an environment in FXS rats despite the reduced temporal compression of representations of these paths. ***C–F***, The distributions for theta sequence properties are shown with shaded areas representing 95% confidence intervals of the distributions for each genotype.

Theta sequences were detected only on days that reached the decoding criterion (see above, Decoding accuracy analysis). Theta sequences were detected and characterized using methods similar to our previously published procedures (Zheng et al., 2016). LFP signals were bandpass filtered in the theta band (6–10 Hz), and individual theta cycles were cut at the theta phase with the lowest number of spikes. Bayesian decoding was then performed on theta cycles in which at least three place cells fired and when the rat was traveling at a speed of 5 cm/s or greater (see above, Bayesian decoding analysis). Bayesian decoding was performed across partially overlapping 40 ms windows advanced by a 10 ms step (Fig. 5*A*,*B*). If spikes did not occur throughout the entirely of the theta cycle, contiguity was considered to be broken when there were two consecutive time bins (i.e., 50 ms) without spikes. Sequence property and significance analyses were then performed on the longest set of contiguous time bins (Fig. 5*C*–*F*). The *t*-span of a sequence was defined as the temporal duration of these time bins. To determine the slope and *x*-span of the sequence event, we calculated the center of mass of the posterior probability distribution *P(x | n)* from the Bayesian decoding for each time bin. A circular–linear regression line was fit to these positions in order to determine the slope. The *x*-span was defined as the distance between the first and last positions of the regression line. To determine if a theta sequence was significant, we compared the *r*^2^ value from this circular–linear regression to a shuffled distribution. This distribution was obtained by circularly shuffling each time bin of the weighted probability distribution [i.e., *P(x | n)* from the Bayesian decoding analysis] a random distance 1,000 times and then obtaining an *r*^2^ value for each shuffled distribution. The *r*^2^ value from the theta sequence had to exceed 95% of the shuffled values in order to be considered significant. Additionally, to ensure the regression provided an accurate representation of the true decoded probability distribution for the event, at least 60% of the total posterior probability needed to be within 20° of the fitted circular–linear regression line (Zheng et al., 2021). In addition, the minimum distance between the fitted trajectory and the actual position of the rat had to be <20° (Zheng et al., 2021). The number of theta sequences from each rat is shown in Table 3.

**Table 3.** Total number of theta sequences recorded from each rat

Genotype	Rat	Number of sequences
WT	326 (“Zachariah”)	3,151
WT	392 (“Danish”)	110
WT	416 (“Desmond”)	1,617
WT	418 (“Hugo”)	2,505
FXS	316 (“Yuki”)	600
FXS	330 (“Aries”)	5,032
FXS	395 (“Elijah”)	461
FXS	442 (“Pippin”)	2,392

### Detection and characterization of replay events

Replay events were detected only on days that reached the decoding criterion (see above, Decoding accuracy analysis). Replay events were detected using methods similar to previously published procedures (Pfeiffer and Foster, 2013; Hwaun and Colgin, 2019). Replay events were detected while the rat rested off of the circular track in a towel-lined flowerpot (see above, Behavior). To detect candidate events, a histogram of population firing rates was constructed using all cells that were classified as active during the track running sessions (see above, Place cell analyses). This histogram was then smoothed with a Gaussian kernel (standard deviation, 10 ms). Candidate events were detected when the population firing rate exceeded three standard deviations above the mean population firing rate and were bounded by crossing of the mean. Events within 40 ms of each other were combined. Start and end times were then adjusted inward so that the first and last time bins of a candidate event each contained at least one spike. To be included for further analysis, an event had to be between 50 and 2,000 ms in duration, and at least five cells had to fire during an event.

**Figure 6.**
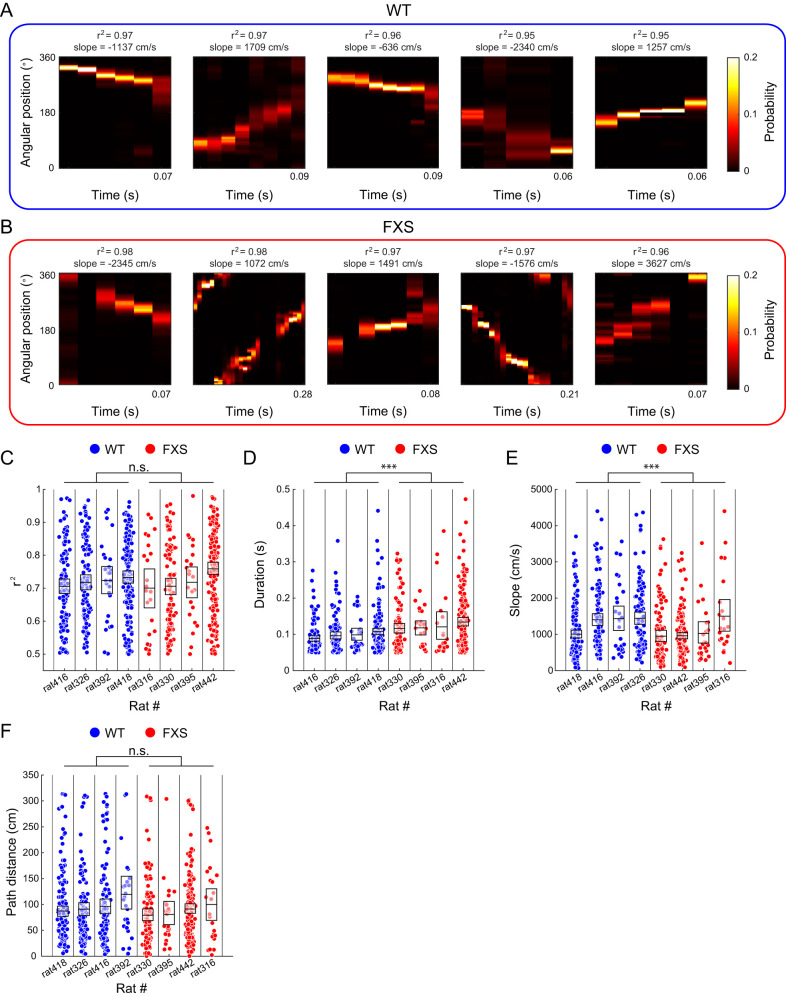
Replay events were less temporally compressed in FXS rats than in WT rats. ***A***, ***B***, Example replay events are shown for WT (***A***) and FXS (***B***) rats. The *r*^2^ value and slope of each replay event are shown above each plot. ***C***, Replay events’ *r*^2^ values did not differ between WT and FXS rats. ***D***, Replay event durations were longer in FXS rats than in WT rats. ***E***, The slopes of the regression lines fit to posterior probability distributions of replay events were lower in FXS rats than in WT rats. ***F***, Path distances of replayed trajectories did not differ between WT and FXS rats. ***C–F***, Each dot represents a measure for one replay event. Boxes represent 95% confidence intervals for the mean values from each rat.

For each candidate event, Bayesian decoding was performed (see above, Bayesian decoding analysis) across partially overlapping 20 ms windows advanced by a 10 ms step (Fig. 6*A*,*B*). To assess how well a posterior probability distribution represented an actual trajectory on the circular track, circular–linear regression was performed with the decoded position as the circular variable and time within the event as the linear variable. The decoded position for each time bin was defined as the center of mass of each time bin, which was determined by taking the circular mean of positions weighted by their associated posterior probability values. The *r*^2^ value of the regression line was used as an assessment of replay fidelity (Fig. 6*C*), as in previous studies (Davidson et al., 2009; Karlsson and Frank, 2009; Hwaun and Colgin, 2019). To be classified as a significant replay event, the *r*^2^ value of an event's regression line had to be at least 0.5 (Hwaun and Colgin, 2019), and the decoded positions between adjacent time bins (i.e., the “jump” distance between adjacent time bins) could not exceed 25% of the length of the circular track (Berners-Lee et al., 2022). Only replay events that occurred after the first track running session of the day (i.e., in Rest Sessions 2–5) were included for further analysis. To quantify the temporal compression of a replay event, we estimated the slope of the circular–linear regression line (Fig. 6*E*). We calculated the absolute value of the slope to include both forward and reverse replay events. The path distance represented within a replay event was calculated as the difference between the decoded positions of the first and last time bins of a replay event (Fig. 6*F*).

### Replay event place cell analyses

**Figure 7.**
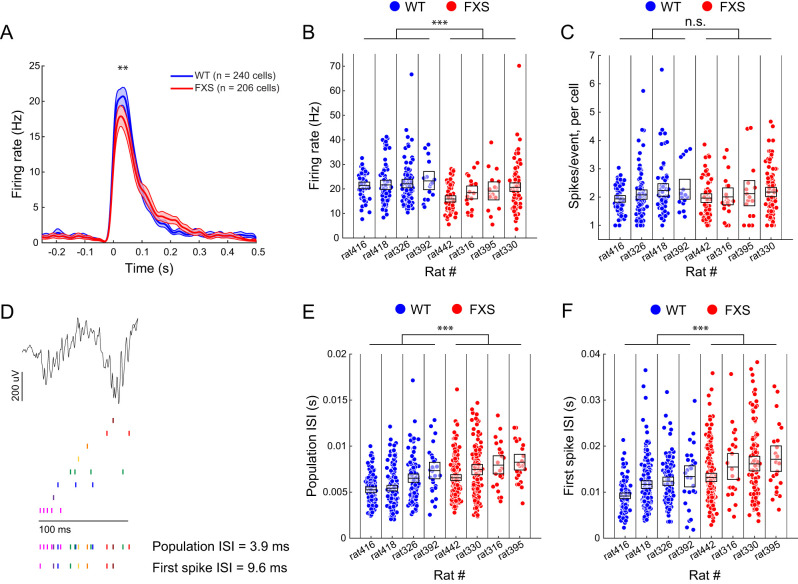
Place cells fired more slowly during replay events in FXS rats than in WT rats. ***A***, Place cells from WT rats reached a higher peak firing rate after replay event onset than place cells from FXS rats. Shaded areas represent 95% confidence intervals of the binned firing rate distributions. ***B***, Place cell firing rates during replay events were higher in WT rats than in FXS rats. ***C***, The number of spikes a place cell fired during replay events did not differ between WT and FXS rats. ***D***, Schematic illustrating how population ISI and first spike ISI were calculated. An LFP recording during an example replay event from a WT rat is shown (top). A raster plot shows spiking activity of eight place cells that participated in the replay event, with spikes from different cells represented by different colored tick marks (middle). The bottom two rows show spikes included when calculating the average ISI for all spikes (population ISI) and only the first spike from each cell (first spike ISI). ***E***, Population ISIs were higher in FXS rats than in WT rats. ***F***, First spike ISIs were higher in FXS rats than in WT rats. ***B***, ***C***, Each dot represents a measure for an individual place cell. Boxes represent 95% confidence intervals for the mean values for each rat. ***E***, ***F***, Each dot represents one replay event. Boxes represent 95% confidence intervals for the mean values for each rat.

To investigate the firing of place cells during replay events, we binned the firing rates of CA1 place cells in 1 ms bins around times of replay event onset for each unit (Hwaun and Colgin, 2019). We only included replay events in which a unit participated (Boone et al., 2018). This allowed us to examine differences in place cell firing patterns during replay events while controlling for differences in the number of replay events in which a unit participates. We smoothed each firing rate vector with a Gaussian kernel (standard deviation, 10 ms). We averaged across all replay events to obtain a binned mean firing rate vector for each unit (Fig. 7*A*). Additionally, we determined the average firing rate for each unit during all replay events in which a unit participated (Fig. 7*B*) and the average number of spikes per event that each unit fired (Fig. 7*C*; Boone et al., 2018).

To determine whether the spike timing of place cells during replay events was impaired in FXS rats, we calculated the interval between spikes in a replay event in two ways (Fig. 7*D*). For the first method, the “population interspike interval (ISI),” we calculated the average interval between consecutive spikes from all cells in a replay event for each replay event (Fig. 7*E*). In the second method, the “first spike ISI,” we only considered the first spike that each cell fired in a replay event and then computed the average interval between consecutive times of first spikes for each replay event (Fig. 7*F*).

### Replay event LFP analyses

**Figure 8.**
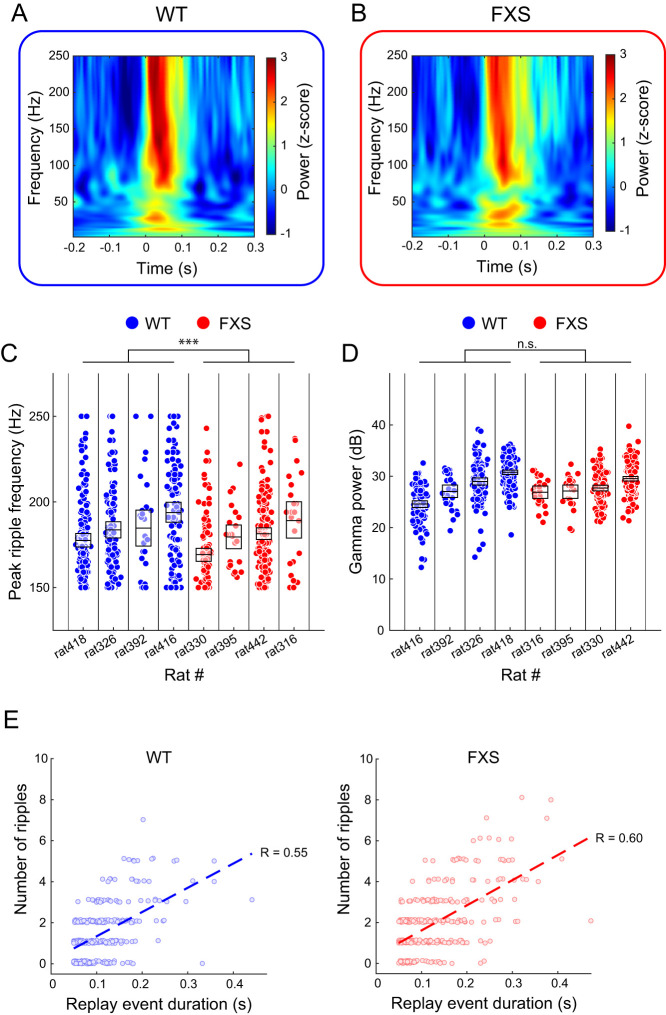
Peak ripple frequency was lower in FXS rats than in WT rats. ***A***, ***B***, Time–frequency representations of power during replay events in WT (***A***) and FXS (***B***) rats. ***C***, Peak ripple frequency was lower in FXS rats than in WT rats. ***D***, Slow gamma power during replay events (25–55 Hz) did not differ between WT and FXS rats. ***C***, ***D***, Each dot represents a measure for one replay event. Boxes represent 95% confidence intervals for the mean values for each rat. ***E***, The number of ripples that co-occurred with replay events of a given duration did not differ between WT (left) and FXS (right) rats. Each dot represents a measure for one replay event. The dashed line represents the regression line fit to the distribution of points for each genotype.

The LFP from the tetrode with the most place cells on a given day was used for each day's analysis of power associated with replay events. The time-varying power across frequencies around the time of replay event onset was computed using a wavelet transform (Tallon-Baudry et al., 1997) as previously described (Mably et al., 2017). Time-varying power around replay event onset was calculated in 1 Hz steps from 2 to 250 Hz and averaged across all replay events within a day. Power was *z*-scored within each frequency band. A time-varying power vector for each frequency was created for each rat by averaging across recording days. The plots presented in Figure 8, *A* and *B*, represent the average time–frequency representations of power associated with replay events across all rats for each genotype. The peak ripple frequency for a replay event (Fig. 8*C*) was defined as the frequency with the highest power in the ripple band, 150–250 Hz. To calculate an overall estimate of slow gamma power associated with a replay event (Fig. 8*D*), we averaged power estimates across the slow gamma band of frequencies (25–55 Hz) and across time for each replay event.

To assess the relationship between the duration of replay events and number of sharp wave-ripples (Fig. 8*E*), we used methods adapted from a previous study (Davidson et al., 2009). The LFP from the tetrode with the most place cells from a given day was bandpass filtered from 150 to 250 Hz, and a Hilbert transform was then applied to the filtered signal. The absolute value of the Hilbert transformed signal was then smoothed with a Gaussian kernel (standard deviation, 8 ms) and *z*-scored. Ripples were detected at times when the *z*-score was ≥3. The number of ripples is displayed with a small amount of random jitter on the *y*-axis for visualization (Fig. 8*E*; Davidson et al., 2009).

### Data visualization

Whenever possible, data are shown for each animal individually. In Figures 2, 3, and 6–8, each data point represents one measure (i.e., for an individual place cell or replay event). Boxes show 95% confidence intervals of the mean values within each rat. Confidence intervals were calculated with 1,000 bootstrapped samples. WT rats are shown with blue data points, and FXS rats are shown with red data points. In each plot for each genotype, data from individual rats were presented in an order corresponding to increasing mean values for ease of comparison across genotypes. In Figures 4 and 5, distributions are shown due to the large number of data points for each genotype. In Figure 5, shaded areas represent 95% confidence intervals calculated with 1,000 bootstrapped samples.

### Experimental design and statistical analyses

Whenever possible, experimenters were blinded to genotype during data acquisition and analyses. However, due to temporarily limited availability of FXS rats from the supplier, experiments on two cohorts of rats were performed unblinded (rats 416, 418, 442, and 445).

MATLAB (MathWorks) scripts were custom written for the analyses in this study, based on algorithms that have been used in prior studies, as described above. All statistical tests were performed using SPSS Statistics (Version 29.0, IBM) unless otherwise stated. To compare behavior, place cell properties, decoding errors, and replay properties across genotypes, we used a generalized linear mixed model design (Robson et al., 2025). Individual rats were subjects, and genotype was included as a fixed factor. When comparing behavior metrics, sessions were nested within days as repeated measures. When applicable, place cells were nested within rats (analyses in Figs. 2, 3, 7*A–C*). When comparing different session pairs to assess place cell firing rate map stability (Fig. 2*A*,*B*), the session pair was included as a repeated measure. To compare decoding errors between genotypes (Fig. 4*C*), we nested the error calculated for each time bin in which a rat was moving faster than 5 cm/s (see above, Decoding accuracy analysis) within sessions, and we nested the sessions within days as repeated measures. For analyses of replay event properties (Figs. 6, 7*E*,*F*, 8*C*,*D*), individual replay events were nested within the rest session in which they occurred, and rest sessions were nested within a day as repeated measures (Boone et al., 2018).

To compare theta sequence properties between genotypes (Fig. 5*C*–*F*), we used the nonparametric Wilcoxon rank-sum test (*ranksum* function in MATLAB).

To account for potential differences in decoding accuracy across rats when analyzing theta sequence slope and replay event slope, we used a generalized linear model (*fitglm* function in MATLAB) with a log-link function. The model included genotype and decoding errors for each session as predictors of slope values to determine if decoding accuracy significantly affected the results (similar to analysis of effects of decoding accuracy in Hwaun and Colgin, 2019).

To assess whether the correlation between replay duration and number of sharp wave-ripples differed across genotypes (Fig. 8*E*), we used multiple linear regression. Genotype, replay event duration, and the interaction between genotype and replay event duration were included as predictors.

### Code and data accessibility

The analysis code is available on GitHub (https://github.com/mmdonahue/FMR1CircTrack_PublicShare). Data will be made available upon reasonable request.

## Results

### Hippocampal place cells showed similar activity in FXS and WT rats

To determine how place cell coding of spatial locations is affected in a rat model of FXS, we recorded from the CA1 pyramidal cell layer of the hippocampus in adult FXS and WT rats. We recorded place cell activity while rats ran unidirectionally on a circular track in a familiar room for four 10 min sessions per day (Fig. 1). WT and FXS rats ran at similar speeds on the circular track (generalized linear mixed model; WT, estimated mean = 34 cm/s; estimated 95% CI = 28–39 cm/s; FXS, estimated mean = 27 cm/s; estimated 95% CI = 22–33 cm/s; no significant main effect of genotype; *F*_(1,98)_ = 2.5; *p* = 0.116), However, FXS rats completed fewer laps per session on average (generalized linear mixed model; WT, estimated mean = 17 laps; estimated 95% CI = 13–22 laps; FXS, estimated mean = 10 laps; estimated 95% CI = 5–15 laps; significant main effect of genotype; *F*_(1,98)_ = 4.4; *p* = 0.039), suggesting that FXS rats paused on the track more than WT rats.

Previous work in a different FXS rat model (i.e., Long–Evans *Fmr1* knock-out rats) found no difference in spatial stability of CA1 place cells when rats explored an initially novel environment over 2 d (Asiminas et al., 2022). However, work from FXS mice reported impaired short-term stability in CA1 place cells (Arbab et al., 2018b). To examine place cell stability in the present FXS rat model, we computed spatial correlation coefficients between pairs of track running sessions for each place cell. Spatial correlations were lower in FXS rats than in WT rats, suggesting impaired place field stability in FXS rats [Fig. 2*A*; generalized linear mixed model, no significant interaction between genotype and session pair (*F*_(3,2,036)_ = 0.399; *p* = 0.754), significant main effect of genotype (*F*_(1,2,036)_ = 4.596; *p* = 0.032)]. Furthermore, place cell firing rates changed more between sessions in FXS rats than in WT rats [Fig. 2*B*; generalized linear mixed model, no significant interaction between genotype and session pair (*F*_(3,2,036)_ = 0.615; *p* = 0.605), significant main effect of genotype (*F*_(1,2,036)_ = 69.542; *p* < 0.001)]. The seeming discrepancy between our results and the relatively stable place fields observed in another rat model of FXS (Asiminas et al., 2022) may be due to differences in testing environments or rat strains.

The previous study of CA1 place cell activity in a different FXS rat model than the one used in the present study showed no impairments in place cells’ spatial information, mean firing rates, or place field size (Asiminas et al., 2022). Here, we also found no differences between FXS and WT rats in CA1 place cells’ spatial information (Fig. 2*C*; generalized linear mixed model, no significant main effect of genotype; *F*_(1,436)_ = 3.674; *p* = 0.056), peak firing rates (Fig. 2*D*; generalized linear mixed model, no significant main effect of genotype; *F*_(1,458)_ = 0.316; *p* = 0.574), and place field size (Fig. 2*E*; generalized linear mixed model, no significant main effect of genotype; *F*_(1,458)_ < 0.001; *p* = 0.995). This indicates that CA1 place cells in FXS rats can represent different locations within a session in a familiar environment as well as CA1 place cells in WT control rats.

### Theta modulation of place cells was normal in FXS rats

During active exploration, the activity of place cells is coordinated by the ∼6–10 Hz theta rhythm in the hippocampus. Theta phase precession refers to a place cell firing pattern in which successive place cell spikes occur at progressively earlier phases of the theta cycle as a rat traverses the cell's place field (O’Keefe and Recce, 1993). Manipulations that reduce theta phase precession (Robbe and Buzsáki, 2009) and disrupt the precise spike timing of neurons during theta sequences (Petersen and Buzsáki, 2020) reduce spatial memory performance, raising the possibility that abnormal theta phase precession contributes to spatial memory deficits that have been reported in FXS models (Bakker et al., 1994; Van Dam et al., 2000; Mineur et al., 2002; Till et al., 2015; Tian et al., 2017; Asiminas et al., 2019). We examined whether CA1 place cells exhibit abnormal phase precession in FXS rats (Fig. 3). We found no difference between FXS and WT rats in the relationship between theta phase and relative position in the place field (Fig. 3*C*; generalized linear mixed model, no significant main effect of genotype; *F*_(1,423)_ = 0.795; *p* = 0.373), suggesting that theta coordination of spiking is normal in FXS rats at the level of individual cells.

### Theta sequences spanned shorter distances in FXS rats

Other work has led to the hypothesis that individual place cells are largely normal in rodent models of FXS while coordinated activity of large populations of place cells is impaired (Talbot et al., 2018). Studies in FXS mice have suggested that hippocampal networks are hypersynchronized in FXS (Talbot et al., 2018; Arbab et al., 2018a). FXS mice also showed abnormally low coordination between place cells with overlapping place fields (Talbot et al., 2018). Together, these prior results suggest that coordinated theta sequences of place cells may be impaired in FXS rats. To assess theta sequences in FXS rats, we used a Bayesian decoding approach (Zhang et al., 1998). Specifically, we applied Bayesian decoding to spiking activity from place cell populations during active running sessions to reconstruct positions on the circular track represented by place cell populations. We first determined how accurately we were able to reconstruct the rat's positions during running using the place cell spiking activity. Most rats’ recordings (WT rats 326, 392, 416, and 418 and FXS rats 316, 330, 395, and 442) surpassed our criteria for sufficient decoding accuracy (Fig. 4*A,B*; see also Materials and Methods, Decoding accuracy analysis). Also, decoding errors were not significantly different between genotypes (Fig. 4*C*; generalized linear mixed model, no significant main effect of genotype; *F*_(1,573,941)_ = 0.17; *p* = 0.678).

For the animals that surpassed decoding accuracy criteria, we used Bayesian decoding to identify trajectories represented by place cell populations within individual theta cycles as WT and FXS rats ran laps unidirectionally on a circular track (Fig. 5*A*,*B*; for the number of theta sequences per rat, see Table 3). We then performed circular–linear regression to fit a regression line to the spatial path represented within these theta cycles. This regression line allowed us to quantify the temporal compression (slope, in cm/s), distance (“*x*-span”, in cm), and duration (“*t*-span”, in s) of each trajectory. We found that the slopes of theta sequences were lower in FXS rats (Fig. 5*C*; Wilcoxon rank-sum test, significant effect of genotype; *Z* = 3.7; *p* < 0.001). To account for potential differences in decoding accuracy across different rats and days, we used a generalized linear model with a log-link function to predict slope values from genotype and the decoding errors for each session. Including genotype in the model increased prediction accuracy while including decoding errors had no significant effect on the model (genotype, *β* = 0.2; *p* = 0.007; decoding accuracy, *β* = 0.08; *p* = 0.32), suggesting that the difference in the temporal compression of theta sequences was not due to differences in decoding accuracy between genotypes. The difference in slope values reflected both a decrease in the distances of paths represented during theta sequences (Fig. 5*D*; Wilcoxon rank-sum test, significant effect of genotype; *Z* = 2.2; *p* = 0.029) and an increase in the duration of events (Fig. 5*E*; Wilcoxon rank-sum test, significant effect of genotype; *Z* = 3.6; *p* < 0.001). We also calculated the *r*^2^ value of the regression line fit to decoded sequence representations in order to assess the extent to which sequences represented coherent trajectories. We found no significant difference in the *r*^2^ values of the circular–linear regression lines for sequences from WT and FXS rats (Fig. 5*F*; Wilcoxon rank-sum test, no significant effect of genotype; *Z* = 1.9; *p* = 0.062). These results suggest that theta sequences represented spatial trajectories in FXS rats. However, theta sequences represented shorter trajectories, and representations were less temporally compressed, in FXS rats compared with WT rats.

### Replay events were less temporally compressed in FXS rats

Replay events co-occur with sharp wave-ripples in the LFP of the hippocampus during NREM sleep or waking rest (Kudrimoti et al., 1999; Nádasdy et al., 1999; Lee and Wilson, 2002; Davidson et al., 2009). Previous work has shown that sharp wave-ripples during NREM sleep are abnormally long in duration in FXS mice (Boone et al., 2018). To assess replay events in FXS rats, we examined the firing of place cell sequences while rats rested quietly in a location off the circular track after running. We used Bayesian decoding to reconstruct positions on the circular track represented by CA1 place cell populations during replay events in WT and FXS rats (Fig. 6*A*,*B*; for the number of replay events recorded from each rat, see Table 4). We quantified replay fidelity, duration, and temporal compression of each replay event using protocols similar to previously published procedures (Davidson et al., 2009; Karlsson and Frank, 2009; Hwaun and Colgin, 2019). Specifically, we first applied circular–linear regression analysis to the posterior probability distributions resulting from Bayesian decoding of place cell spikes during replay events. We then assessed associated *r*^2^ values as a measure of replay fidelity, and the slopes of the fitted lines were used to estimate the temporal compression of replay events. There was no difference in replay fidelity between WT and FXS rats (Fig. 6*C*; generalized linear mixed model, no significant effect of genotype; *F*_(1,751)_ = 2.185; *p* = 0.140). However, the duration of replay events was longer in FXS rats than in WT rats (Fig. 6*D*; generalized linear mixed model, significant effect of genotype; *F*_(1,751)_ = 33.332; *p* < 0.001). Furthermore, the representations during replay events were less temporally compressed, indicating slower transitions across representations of successive locations, in FXS rats than in WT rats (Fig. 6*E*; generalized linear mixed model, significant effect of genotype; *F*_(1,751)_ = 19.340; *p* < 0.001). To account for potential differences in decoding accuracy across different rats and days, we used a generalized linear model with a log-link function to predict slope values from genotype and the decoding errors for each session. Including genotype in the model increased prediction accuracy while including decoding errors had no significant effect on the model (genotype, *β* = 0.25; *p* < 0.001; decoding accuracy, *β* = 0.06; *p* = 0.82), suggesting that the difference in temporal compression of replay events was not due to different decoding accuracy between genotypes. The low temporal compression in FXS rats did not indicate that replay events represented unusually long paths in FXS rats, however, because path lengths of replay sequences were similar in FXS and WT rats (Fig. 6*F*; generalized linear mixed model, no significant effect of genotype; *F*_(1,751)_ = 1.3; *p* = 0.253). Instead, this pattern of results suggests that replay events in FXS rats represented trajectories of similar lengths and comparable fidelity as in WT rats but that replay of representations of trajectories occurred more slowly in FXS rats than in WT rats.

**Table 4.** Total number of replay events from each rat

Genotype	Rat	Number of replay events
WT	326 (“Zachariah”)	114
WT	392 (“Danish”)	28
WT	416 (“Desmond”)	116
WT	418 (“Hugo”)	166
FXS	316 (“Yuki”)	23
FXS	330 (“Aries”)	110
FXS	395 (“Elijah”)	24
FXS	442 (“Pippin”)	172

### Place cells fired more slowly during replay events in FXS rats

In addition to differences in duration, Boone and colleagues (Boone et al., 2018) observed abnormal place cell firing during sharp wave-ripples in a mouse model of FXS. They found that place cells had lower in-event firing rates during sharp wave-ripples recorded during sleep, although individual cells fired the same number of spikes per event (Boone et al., 2018). Abnormally slow firing of place cells during replay events in FXS rats may underlie the reduced temporal compression observed in our data. Indeed, we found that CA1 place cells had lower peak firing rates after replay event onset in FXS rats than in WT rats (Fig. 7*A*; generalized linear mixed model, significant effect of genotype on peak firing rate after event onset; *F*_(1,444)_ = 8.877; *p* = 0.003). In-event firing rates were also lower in FXS rats than in WT rats (Fig. 7*B*; generalized linear mixed model, significant effect of genotype; *F*_(1,444)_ = 23.140; *p* < 0.001), while the number of spikes each cell fired during a replay event did not differ between FXS and WT rats (Fig. 7*C*; generalized linear mixed model, no significant effect of genotype; *F*_(1,444)_ = 1.020; *p* = 0.313) likely because of replay events’ relatively long durations in FXS rats (Fig. 6*D*). The low replay-associated firing rates in individual place cells in FXS rats suggest that dynamics of CA1 place cell sequence firing may occur more slowly during replay events in FXS rats than in WT rats. To test this hypothesis, we calculated the distribution of intervals between successive spikes in a replay event in two ways (Fig. 7*D*). First, we considered all spikes that occurred across all cells during a replay event and found that these population ISIs were longer in FXS rats than in WT rats (Fig. 7*E*; generalized linear mixed model, significant effect of genotype; *F*_(1,751)_ = 58.337; *p* < 0.001). Next, we only considered the first spike that each cell in a sequence fired in a replay event (Fig. 7*F*). These first spike ISIs were also longer in FXS rats than in WT rats (generalized linear mixed model, significant effect of genotype; *F*_(1,751)_ = 56.843; *p* < 0.001).

### Properties of ripples and slow gamma rhythms during replay events in FXS rats

We further examined properties of oscillatory patterns in the LFP, specifically sharp wave-ripples and slow gamma oscillations, that normally co-occur with replay events in WT rats (Kudrimoti et al., 1999; Nádasdy et al., 1999; Lee and Wilson, 2002; Davidson et al., 2009; Carr et al., 2012; Bieri et al., 2014). In both genotypes, we saw increases in power in the ripple and slow gamma bands at the event onset (Fig. 8*A*,*B*), suggesting that ripples and slow gamma rhythms co-occur with replay events in FXS rats as in WT rats. However, the peak ripple frequency during replay events was lower in FXS rats than in WT rats (Fig. 8*C*; generalized linear mixed model, significant effect of genotype; *F*_(1,751)_ = 21.168; *p* < 0.001), consistent with results from FXS mice showing lower peak ripple frequency during sharp wave-ripples during sleep (Boone et al., 2018). Although the frequency of ripples was lower in FXS rats, the number of sharp wave-ripples that co-occurred with a replay event of a given duration was similar in FXS and WT rats [Fig. 8*E*; significant multiple linear regression (*F*_(3,749)_ = 145.149; *p* < 0.001); no significant interaction between genotype and replay event duration (*t* = 0.369; *p* = 0.712)], likely due to the longer duration of replay events in FXS rats than in WT rats (Fig. 6*D*).

Previous work has shown increased slow gamma power in CA1 during sleep-associated sharp wave-ripples in a FXS mouse model (Boone et al., 2018). Here, we found no difference in slow gamma power during rest-associated replay events in WT and FXS rats (Fig. 8*D*; generalized linear mixed model, no significant effect of genotype; *F*_(1,751)_ = 0.095; *p* = 0.758). Replay events during sleep and waking rest may have different functions and characteristics (Roumis and Frank, 2015; Tang et al., 2017) and may involve input from different upstream structures to CA1 (Yamamoto and Tonegawa, 2017). Thus, differences in rest and sleep states may underlie the seemingly discrepant results for replay-associated slow gamma in FXS rat and mouse models.

## Discussion

To our knowledge, this is the first study that examines spatial representations coded by large populations of hippocampal place cells in a rodent model of FXS. Here, we present data showing that coding of spatial trajectories by coordinated place cell populations is impaired in FXS rats. Specifically, we found that theta sequences coded less temporally compressed representations and represented shorter path distances in FXS rats, while theta phase precession in individual place cells was normal. Furthermore, we found that place cell sequences fired abnormally slowly during hippocampal replay events in FXS rats. This slow place cell firing was associated with reduced temporal compression of representations of track trajectories during replay in FXS rats. Together, these results suggest that coordinated place cell sequences code spatial trajectories abnormally during both active running and during subsequent rest in a rat model of FXS.

Previous studies examining the activity of place cells during active exploration in FXS rodents have yielded mixed results. Studies from FXS mice and rats have shown no difference in spatial information in CA1 place cells (Talbot et al., 2018; Arbab et al., 2018b; Asiminas et al., 2022), although one study has shown abnormally large place field sizes and excessive out-of-field firing in FXS mice (Arbab et al., 2018b). Discrepancies between these results and the present findings may result from different experimental paradigms, including different testing arenas, experimental time courses, and the degree of environmental novelty. Regarding the latter point, work from FXS rats has shown that experience-dependent reductions in firing rates and sharpening of spatial tuning of place cells are lacking in FXS rats after introduction to a novel environment (Asiminas et al., 2022). These results suggest that the initial formation of a spatial memory may be stunted in FXS rats. We did not observe such differences in our study, likely due to our use of a testing environment with which rats had already been familiarized.

Work from FXS mice has shown normal theta modulation in CA1 place cells but reduced correlated firing of pairs of place cells (Talbot et al., 2018). This suggested that coordinated population coding would be impaired in FXS rodents. Consistent with this result, we show that theta-coordinated sequences of place cells during active exploration represented shorter trajectories in FXS rats, while theta phase precession in individual place cells remained normal. While theta phase precession and theta sequences appear to be related phenomena, it is possible to observe the absence of theta sequences in populations of place cells that individually show intact phase precession (Feng et al., 2015; Middleton and McHugh, 2016). Computational models have suggested that alterations in the coupling between place cells and local CA1 interneurons may disrupt the temporal compression of theta sequences while leaving phase precession intact (Chadwick et al., 2016). Interneurons are less modulated by theta in FXS mice (Talbot et al., 2018), and CA1 interneurons and pyramidal cells are less correlated in FXS mice (Arbab et al., 2018a). This interneuron–pyramidal cell dysfunction may result in hypersynchronization between CA1 pyramidal neurons and thereby cause place cells that represent multiple locations along a trajectory to fire more synchronously in FXS rats. This could flatten slopes of theta sequences and shorten distances of represented trajectories.

Disrupting the input from CA3 to CA1 may also affect the path length of theta sequences (Chadwick et al., 2016; Middleton and McHugh, 2016). In vitro work examining the Schaffer collateral pathway from CA3 to CA1 in FXS has yielded mixed results. Work from FXS mice has shown an abnormally low induction threshold for long-term potentiation (LTP) in FXS mice when pre- and postsynaptic neurons are simultaneously activated (Routh et al., 2013). However, other works from FXS mice (Lauterborn et al., 2007) and rats (Tian et al., 2017) have reported reduced Schaffer collateral LTP. FXS mouse models have also shown increased metabotropic glutamate receptor long-term depression (Huber et al., 2002; Zhang et al., 2009; Iliff et al., 2013), suggesting that hippocampal synapses may be relatively weak in rodent models of FXS. Such a reduction in synaptic input from CA3 to CA1 may affect the temporal compression of theta sequences in FXS rats, resulting in representation of relatively short paths.

Our work shows that CA1 place cells fired more slowly in FXS rats than in WT rats during replay events that occurred during waking rest. This finding is consistent with abnormally slow firing of pyramidal cells during sharp wave-ripples in non-REM sleep reported in FXS mice (Boone et al., 2018). In vitro work from FXS rats has also shown lower-frequency multiunit activity during sharp wave-ripples specifically in the dorsal hippocampus (Leontiadis et al., 2023). Parvalbumin-expressing inhibitory interneurons in the hippocampus are important for pacing pyramidal cell spiking during sharp wave-ripples (Schlingloff et al., 2014; Stark et al., 2014), and hippocampal network models have suggested that inhibition within CA1 is important for controlling cell participation within replay events (Ramirez-Villegas et al., 2018). Reported weaknesses in pyramidal cell–interneuron coupling in CA1 have only been examined during active behavior in FXS models (Arbab et al., 2018a). However, considering that ripple oscillations are locally generated in CA1 (Csicsvari et al., 1999), the lower peak ripple frequencies that we found during replay events may suggest that firing of CA1 inhibitory interneurons is also slowed during ripples in FXS rats. Due to the limited number of interneurons in our dataset, we were not able to test this hypothesis directly in the present study.

Both CA3 and CA2 contribute to generation of sharp wave-ripples and replay events in CA1 (Csicsvari et al., 1999, 2000; Oliva et al., 2016). Thus, deficits in CA1 place cell firing in FXS rats during replay events may be inherited from these upstream regions or due to local deficits in CA1. Future studies involving simultaneous recordings from CA3, CA2, and CA1 are necessary to shed light on circuit mechanisms underlying slowed firing of place cells during replay events in FXS rats.

In addition to inputs to CA1 from CA3 and CA2, the input from the medial entorhinal cortex (MEC) to CA1 can be important for replay events, particularly replay events that span multiple sharp wave-ripples (Yamamoto and Tonegawa, 2017). Previous work has suggested that synaptic inputs from MEC to CA1 pyramidal cells are reduced in FXS models (Ordemann et al., 2021; Asiminas et al., 2022). Although we did not observe a difference in the number of ripples co-occurring with replay events of a given duration between FXS and WT rats (Fig. 8*E*), a reduction in MEC input during replay events may affect temporal compression of replayed sequences for extended replay events in CA1.

Downstream targets of the hippocampus may be affected by impaired temporal compression of awake replay events. During replay events, activity between the hippocampus and prefrontal cortex is coordinated (Peyrache et al., 2009; Jadhav et al., 2016; Tang et al., 2017; Shin et al., 2019; Berners-Lee et al., 2021; Harvey et al., 2023). This coordinated activity has been hypothesized to support memory retrieval processes that can be used to guide future decision-making (Jadhav et al., 2016; Zielinski et al., 2020). The strength of excitatory drive from the hippocampus to the prefrontal cortex during sharp wave-ripples can affect the response of the prefrontal cortex (Wierzynski et al., 2009), suggesting that abnormally slowed spike timing during replay events in CA1 of FXS rats may alter subsequent prefrontal responses.

The work presented shows novel evidence for specific physiological impairments in the hippocampus in a rat model of FXS. However, these impairments were characterized using a simple behavioral protocol with no memory component. Rats were familiarized to the environment before recording, and food rewards were randomly given without any motivational salience for specific spatial trajectories. Previous work suggests that the temporal compression of place cell sequences during both active behavior and awake rest can contribute to spatial learning and memory. The slopes and strength of theta sequences have been reported to increase during learning of new environments or trajectories to a new goal location (Feng et al., 2015; Igata et al., 2021; Pfeiffer, 2022), and theta sequences exhibited higher slopes during correct trials than error trials of a spatial memory task (Zheng et al., 2021). Replay duration and temporal compression have been linked to learning and memory in studies of healthy WT rats (Fernández-Ruiz et al., 2019; Berners-Lee et al., 2022). Replay duration increased, and temporal compression of replay events decreased, across the first several paths that rats took during learning of a new environment (Berners-Lee et al., 2022). However, on a longer timescale, the duration of replay events decreased, while the lengths of trajectory representations increased, across sessions in rats learning a spatial memory task (Shin et al., 2019). These results raise the possibility that less temporally compressed theta sequences and slow replay could contribute to impaired spatial learning and memory in FXS rats. Future studies of place cell population activity in FXS rats engaged in learning and memory tasks will be important to shed light on this question.

## References

[B1] Arbab T, Battaglia FP, Pennartz CMA, Bosman CA (2018a) Abnormal hippocampal theta and gamma hypersynchrony produces network and spike timing disturbances in the Fmr1-KO mouse model of Fragile X syndrome. Neurobiol Dis 114:65–73. 10.1016/j.nbd.2018.02.011 29486296

[B2] Arbab T, Pennartz CMA, Battaglia FP (2018b) Impaired hippocampal representation of place in the Fmr1-knockout mouse model of fragile X syndrome. Sci Rep 8:1–9. 10.1038/s41598-018-26853-z 29892074 PMC5995880

[B3] Asiminas A, et al. (2019) Sustained correction of associative learning deficits after brief, early treatment in a rat model of Fragile X syndrome. Sci Transl Med 11:1–10. 10.1126/scitranslmed.aao0498 31142675 PMC8162683

[B4] Asiminas A, Booker SA, Dando OR, Kozic Z, Arkell D, Inkpen FH, Sumera A, Akyel I, Kind PC, Wood ER (2022) Experience-dependent changes in hippocampal spatial activity and hippocampal circuit function are disrupted in a rat model of Fragile X syndrome. Mol Autism 13:1–29. 10.1186/s13229-022-00528-z 36536454 PMC9764562

[B5] Bakker CE, et al. (1994) Fmr1 knockout mice: a model to study fragile X mental retardation. Cell 78:23–33. 10.1016/0092-8674(94)90569-X 8033209

[B6] Berens P (2009) Circstat: a MATLAB toolbox for circular statistics. J Stat Softw 31:1–21. 10.18637/jss.v031.i10

[B7] Berners-Lee A, Feng T, Silva D, Wu X, Ambrose ER, Pfeiffer BE, Foster DJ (2022) Hippocampal replays appear after a single experience and incorporate greater detail with more experience. Neuron 110:1829–1842.e5. 10.1016/j.neuron.2022.03.010 35381188 PMC9514662

[B8] Berners-Lee A, Wu X, Foster DJ (2021) Prefrontal cortical neurons are selective for non-local hippocampal representations during replay and behavior. J Neurosci 41:5894–5908. 10.1523/JNEUROSCI.1158-20.2021 34035138 PMC8265798

[B9] Bieri KW, Bobbitt KN, Colgin LL (2014) Slow and fast gamma rhythms coordinate different spatial coding modes in hippocampal place cells. Neuron 82:670–681. 10.1016/j.neuron.2014.03.013 24746420 PMC4109650

[B10] Boone CE, Davoudi H, Harrold JB, Foster DJ (2018) Abnormal sleep architecture and hippocampal circuit dysfunction in a mouse model of Fragile X syndrome. Neuroscience 384:275–289. 10.1016/j.neuroscience.2018.05.012 29775702

[B11] Buzsáki G (2002) Theta oscillations in the hippocampus. Neuron 33:325–340. 10.1016/S0896-6273(02)00586-X 11832222

[B12] Carr MF, Karlsson MP, Frank LM (2012) Transient slow gamma synchrony underlies hippocampal memory replay. Neuron 75:700–713. 10.1016/j.neuron.2012.06.014 22920260 PMC3428599

[B13] Chadwick A, Van Rossum MCW, Nolan MF (2016) Flexible theta sequence compression mediated via phase precessing interneurons. Elife 5:e20349. 10.7554/ELIFE.20349 27929374 PMC5245972

[B14] Colgin LL, Leutgeb S, Jezek K, Leutgeb JK, Moser EI, McNaughton BL, Moser MB (2010) Attractor-map versus autoassociation based attractor dynamics in the hippocampal network. J Neurophysiol 104:35–50. 10.1152/jn.00202.2010 20445029 PMC2904215

[B15] Cornish KM, Munir F, Cross G (1998) The nature of the spatial deficit in young females with Fragile-X syndrome: a neuropsychological and molecular perspective. Neuropsychologia 36:1239–1246. 10.1016/S0028-3932(97)00162-0 9842768

[B16] Csicsvari J, Hirase H, Czurkó A, Mamiya A, Buzsáki G (1999) Fast network oscillations in the hippocampal CA1 region of the behaving rat. J Neurosci 19:RC20. 10.1523/jneurosci.19-16-j0001.1999 10436076 PMC6782850

[B17] Csicsvari J, Hirase H, Mamiya A, Buzsáki G (2000) Ensemble patterns of hippocampal CA3-CA1 neurons during sharp wave-associated population events. Neuron 28:585–594. 10.1016/S0896-6273(00)00135-5 11144366

[B18] Darnell JC, et al. (2011) FMRP stalls ribosomal translocation on mRNAs linked to synaptic function and autism. Cell 146:247–261. 10.1016/j.cell.2011.06.013 21784246 PMC3232425

[B19] Darnell JC, Klann E (2013) The translation of translational control by FMRP: therapeutic targets for FXS. Nat Neurosci 16:1530–1536. 10.1038/nn.3379 23584741 PMC3999698

[B20] Davidson TJ, Kloosterman F, Wilson MA (2009) Hippocampal replay of extended experience. Neuron 63:497–507. 10.1016/j.neuron.2009.07.027 19709631 PMC4364032

[B21] De Boulle K, Verkerk AJMH, Reyniers E, Vits L, Hendrickx J, Van Roy B, Van Den Bos F, de Graaff E, Oostra BA, Willems PJ (1993) A point mutation in the FMR-1 gene associated with fragile X mental retardation. Nat Genet 3:31–35. 10.1038/ng0193-31 8490650

[B22] Feng T, Silva D, Foster DJ (2015) Dissociation between the experience-dependent development of hippocampal theta sequences and single-trial phase precession. J Neurosci 35:4890–4902. 10.1523/JNEUROSCI.2614-14.2015 25810520 PMC4389593

[B23] Fernández-Ruiz A, Oliva A, de Oliveira EF, Rocha-Almeida F, Tingley D, Buzsáki G (2019) Long-duration hippocampal sharp wave ripples improve memory. Science 364:1082–1086. 10.1126/science.aax0758 31197012 PMC6693581

[B24] Foster DJ, Wilson MA (2007) Hippocampal theta sequences. Hippocampus 17:1093–1099. 10.1002/hipo.20345 17663452

[B25] Gallagher A, Hallahan B (2012) Fragile X-associated disorders: a clinical overview. J Neurol 259:401–413. 10.1007/s00415-011-6161-3 21748281

[B26] Harvey RE, Robinson HL, Liu C, Oliva A, Fernandez-Ruiz A (2023) Hippocampo-cortical circuits for selective memory encoding, routing, and replay. Neuron 111:2076–2090.e9. 10.1016/j.neuron.2023.04.015 37196658 PMC11146684

[B27] Hollup SA, Molden S, Donnett JG, Moser MB, Moser EI (2001) Accumulation of hippocampal place fields at the goal location in an annular watermaze task. J Neurosci 21:1635–1644. 10.1523/jneurosci.21-05-01635.2001 11222654 PMC6762966

[B28] Hsiao YT, Zheng C, Colgin LL (2016) Slow gamma rhythms in CA3 are entrained by slow gamma activity in the dentate gyrus. J Neurophysiol 116:2594–2603. 10.1152/jn.00499.2016 27628206 PMC5133296

[B29] Huber KM, Gallagher SM, Warren ST, Bear MF (2002) Altered synaptic plasticity in a mouse model of fragile X mental retardation. Proc Natl Acad Sci U S A 99:7746–7750. 10.1073/PNAS.122205699/ASSET/4D74A266-B9D1-4EEC-A089-AB9F614C8070/ASSETS/GRAPHIC/PQ1222056004.JPEG 12032354 PMC124340

[B30] Hwaun E, Colgin LL (2019) CA3 place cells that represent a novel waking experience are preferentially reactivated during sharp wave-ripples in subsequent sleep. Hippocampus 29:921–938. 10.1002/hipo.23090 30891854 PMC7412067

[B31] Igata H, Ikegaya Y, Sasaki T (2021) Prioritized experience replays on a hippocampal predictive map for learning. Proc Natl Acad Sci U S A 118:e20112. 10.1073/PNAS.2011266118/SUPPL_FILE/PNAS.2011266118.SM02.MP4 PMC7817193 33443144

[B32] Iliff AJ, Renoux AJ, Krans A, Usdin KU, Sutton MA, Todd PK (2013) Impaired activity-dependent FMRP translation and enhanced mGluR-dependent LTD in Fragile X premutation mice. Hum Mol Genet 22:1180–1192. 10.1093/HMG/DDS525 23250915 PMC3578412

[B33] Jadhav SP, Rothschild G, Roumis DK, Frank LM (2016) Coordinated excitation and inhibition of prefrontal ensembles during awake hippocampal sharp-wave ripple events. Neuron 90:113–127. 10.1016/j.neuron.2016.02.010 26971950 PMC4824654

[B34] Jäkälä P, Hänninen T, Ryynänen M, Laakso M, Partanen K, Mannermaa A, Soininen H (1997) Fragile-X: neuropsychological test performance, CGG triplet repeat lengths, and hippocampal volumes. J Clin Invest 100:331–338. 10.1172/JCI119538 9218509 PMC508195

[B35] Karlsson MP, Frank LM (2009) Awake replay of remote experiences in the hippocampus. Nat Neurosci 12:913–918. 10.1038/nn.2344 19525943 PMC2750914

[B36] Kremer EJ, Pritchard M, Lynch M, Yu S, Holman K, Baker E, Warren ST, Schlessinger D, Sutherland GR, Richards RI (1991) Mapping of DNA instability at the fragile X to a trinucleotide repeat sequence p(CCG)n. Science 252:1711–1714. 10.1126/science.1675488 1675488

[B37] Kudrimoti HS, Barnes CA, McNaughton BL (1999) Reactivation of hippocampal cell assemblies: effects of behavioral state, experience, and EEG dynamics. J Neurosci 19:4090–4101. 10.1523/jneurosci.19-10-04090.1999 10234037 PMC6782694

[B38] Lauterborn JC, Rex CS, Kramár E, Chen LY, Pandyarajan V, Lynch G, Gall CM (2007) Brain-derived neurotrophic factor rescues synaptic plasticity in a mouse model of Fragile X syndrome. J Neurosci 27:10685–10694. 10.1523/JNEUROSCI.2624-07.2007 17913902 PMC6672822

[B39] Lee AK, Wilson MA (2002) Memory of sequential experience in the hippocampus during slow wave sleep. Neuron 36:1183–1194. 10.1016/S0896-6273(02)01096-6 12495631

[B40] Leontiadis LJ, Trompoukis G, Tsotsokou G, Miliou A, Felemegkas P, Papatheodoropoulos C (2023) Rescue of sharp wave-ripples and prevention of network hyperexcitability in the ventral but not the dorsal hippocampus of a rat model of fragile X syndrome. Front Cell Neurosci 17:1296235. 10.3389/FNCEL.2023.1296235/BIBTEX 38107412 PMC10722241

[B41] Ludwig AL, Espinal GM, Pretto DI, Jamal AL, Arque G, Tassone F, Berman RF, Hagerman PJ (2014) CNS expression of murine fragile X protein (FMRP) as a function of CGG-repeat size. Hum Mol Genet 23:3228–3238. 10.1093/HMG/DDU032 24463622 PMC4030777

[B42] Mably AJ, Gereke BJ, Jones DT, Colgin LL (2017) Impairments in spatial representations and rhythmic coordination of place cells in the 3xTg mouse model of Alzheimer’s disease. Hippocampus 27:378–392. 10.1002/hipo.22697 28032686

[B43] Machalicek W, McDuffie A, Oakes A, Ma M, Thurman AJ, Rispoli MJ, Abbeduto L (2014) Examining the operant function of challenging behavior in young males with fragile X syndrome: a summary of 12 cases. Res Dev Disabil 35:1694–1704. 10.1016/j.ridd.2014.03.014 24679547

[B44] Middleton SJ, McHugh TJ (2016) Silencing CA3 disrupts temporal coding in the CA1 ensemble. Nat Neurosci 19:945–951. 10.1038/nn.4311 27239937

[B45] Mikiko CS, Siomi H, Sauer WH, Srinivasan S, Nussbaum RL, Dreyfuss G (1995) FXR1, an autosomal homolog of the fragile X mental retardation gene. EMBO J 14:2401–2408. 10.1002/j.1460-2075.1995.tb07237.x 7781595 PMC398353

[B46] Mineur YS, Sluyter F, De Wit S, Oostra BA, Crusio WE (2002) Behavioral and neuroanatomical characterization of the Fmr1 knockout mouse. Hippocampus 12:39–46. 10.1002/hipo.10005 11918286

[B47] Muller K, Brady NC, Warren SF, Fleming KK (2019) Mothers’ perspectives on challenging behaviours in their children with fragile X syndrome. J Intellect Dev Disabil 44:481–491. 10.3109/13668250.2018.1496379 31896952 PMC6939860

[B48] Nádasdy Z, Hirase H, Czurkó A, Csicsvari J, Buzsáki G (1999) Replay and time compression of recurring spike sequences in the hippocampus. J Neurosci 19:9497–9507. 10.1523/jneurosci.19-21-09497.1999 10531452 PMC6782894

[B49] O’Keefe J (1976) Place units in the hippocampus of the freely moving rat. Exp Neurol 51:78–109. 10.1016/0014-4886(76)90055-8 1261644

[B50] O’Keefe J, Dostrovsky J (1971) The hippocampus as a spatial map. Preliminary evidence from unit activity in the freely-moving rat. Brain Res 34:171–175. 10.1016/0006-8993(71)90358-1 5124915

[B51] O’Keefe J, Recce ML (1993) Phase relationship between hippocampal place units and the EEG theta rhythm. Hippocampus 3:317–330. 10.1002/hipo.450030307 8353611

[B52] Oliva A, Fernández-Ruiz A, Buzsáki G, Berényi A (2016) Role of hippocampal CA2 region in triggering sharp-wave ripples. Neuron 91:1342–1355. 10.1016/j.neuron.2016.08.008 27593179 PMC8138857

[B53] Ordemann GJ, Apgar CJ, Chitwood RA, Brager DH (2021) Altered A-type potassium channel function impairs dendritic spike initiation and temporoammonic long-term potentiation in Fragile X syndrome. J Neurosci 41:5947–5962. 10.1523/JNEUROSCI.0082-21.2021 34083256 PMC8265803

[B54] Petersen PC, Buzsáki G (2020) Cooling of medial septum reveals theta phase lag coordination of hippocampal cell assemblies. Neuron 107:731–744.e3. 10.1016/j.neuron.2020.05.023 32526196 PMC7442698

[B55] Peyrache A, Khamassi M, Benchenane K, Wiener SI, Battaglia FP (2009) Replay of rule-learning related neural patterns in the prefrontal cortex during sleep. Nat Neurosci 12:919–926. 10.1038/nn.2337 19483687

[B56] Pfeiffer BE (2022) Spatial learning drives rapid goal representation in hippocampal ripples without place field accumulation or goal-oriented theta sequences. J Neurosci 42:3975–3988. 10.1523/JNEUROSCI.2479-21.2022 35396328 PMC9097771

[B57] Pfeiffer BE, Foster DJ (2013) Hippocampal place-cell sequences depict future paths to remembered goals. Nature 497:74–79. 10.1038/nature12112 23594744 PMC3990408

[B58] Pieretti M, Zhang F, Fu YH, Warren ST, Oostra BA, Caskey CT, Nelson DL (1991) Absence of expression of the FMR-1 gene in fragile X syndrome. Cell 66:817–822. 10.1016/0092-8674(91)90125-I 1878973

[B59] Ramirez-Villegas JF, Willeke KF, Logothetis NK, Besserve M (2018) Dissecting the synapse- and frequency-dependent network mechanisms of in vivo hippocampal sharp wave-ripples. Neuron 100:1224–1240.e13. 10.1016/j.neuron.2018.09.041 30482688

[B60] Robbe D, Buzsáki G (2009) Alteration of theta timescale dynamics of hippocampal place cells by a cannabinoid is associated with memory impairment. J Neurosci 29:12597–12605. 10.1523/JNEUROSCI.2407-09.2009 19812334 PMC2799373

[B61] Robson E, Donahue MM, Mably AJ, Demetrovich PG, Hewitt LT, Colgin LL (2025) Social odors drive hippocampal CA2 place cell responses to social stimuli. Prog Neurobiol 245:102708. 10.1016/j.pneurobio.2024.102708 39743170 PMC11827691

[B62] Roumis DK, Frank LM (2015) Hippocampal sharp-wave ripples in waking and sleeping states. Curr Opin Neurobiol 35:6–12. 10.1016/j.conb.2015.05.001 26011627 PMC4641767

[B63] Routh BN, Johnston D, Brager DH (2013) Loss of functional a-type potassium channels in the dendrites of CA1 pyramidal neurons from a mouse model of Fragile X syndrome. J Neurosci 33:19442–19450. 10.1523/JNEUROSCI.3256-13.2013 24336711 PMC3858620

[B64] Schlingloff D, Káli S, Freund TF, Hájos N, Gulyás AI (2014) Mechanisms of sharp wave initiation and ripple generation. J Neurosci 34:11385–11398. 10.1523/JNEUROSCI.0867-14.2014 25143618 PMC6615505

[B65] Shin JD, Tang W, Jadhav SP (2019) Dynamics of awake hippocampal-prefrontal replay for spatial learning and memory-guided decision making. Neuron 104:1110–1125.e7. 10.1016/j.neuron.2019.09.012 31677957 PMC6923537

[B66] Siomi H, Siomi MC, Nussbaum RL, Dreyfuss G (1993) The protein product of the fragile X gene, FMR1, has characteristics of an RNA-binding protein. Cell 74:291–298. 10.1016/0092-8674(93)90420-U 7688265

[B67] Skaggs WE, McNaughton BL, Wilson MA, Barnes CA (1996) Theta phase precession in hippocampal neuronal populations and the compression of temporal sequences. Hippocampus 6:149–172. 10.1002/(SICI)1098-1063(1996)6:2<149::AID-HIPO6>3.0.CO;2-K 8797016

[B68] Stark E, Roux L, Eichler R, Senzai Y, Royer S, Buzsáki G (2014) Pyramidal cell-interneuron interactions underlie hippocampal ripple oscillations. Neuron 83:467–480. 10.1016/j.neuron.2014.06.023 25033186 PMC4393648

[B69] Talbot ZN, Sparks FT, Dvorak D, Curran BM, Alarcon JM, Fenton AA (2018) Normal CA1 place fields but discoordinated network discharge in a Fmr1-null mouse model of Fragile X syndrome. Neuron 97:684–697.e4. 10.1016/j.neuron.2017.12.043 29358017 PMC6066593

[B70] Tallon-Baudry C, Bertrand O, Delpuech C, Pernier J (1997) Oscillatory γ-band (30–70Hz) activity induced by a visual search task in humans. J Neurosci 17:722–734. 10.1523/JNEUROSCI.17-02-00722.1997 8987794 PMC6573221

[B71] Tang W, Shin JD, Frank LM, Jadhav SP (2017) Hippocampal-prefrontal reactivation during learning is stronger in awake compared with sleep states. J Neurosci 37:11789–11805. 10.1523/JNEUROSCI.2291-17.2017 29089440 PMC5719968

[B72] Tian Y, et al. (2017) Loss of FMRP impaired hippocampal long-term plasticity and spatial learning in rats. Front Mol Neurosci 10:269. 10.3389/fnmol.2017.00269 28894415 PMC5581399

[B73] Till SM, et al. (2015) Conserved hippocampal cellular pathophysiology but distinct behavioural deficits in a new rat model of FXS. Hum Mol Genet 24:5977–5984. 10.1093/HMG/DDV299 26243794 PMC4599667

[B74] Van Dam D, D’Hooge R, Hauben E, Reyniers E, Gantois I, Bakker CE, Oostra BA, Kooy RF, De Deyn PP (2000) Spatial learning, contextual fear conditioning and conditioned emotional response in Fmr1 knockout mice. Behav Brain Res 117:127–136. 10.1016/S0166-4328(00)00296-5 11099766

[B75] Wierzynski CM, Lubenov EV, Gu M, Siapas AG (2009) State-dependent spike-timing relationships between hippocampal and prefrontal circuits during sleep. Neuron 61:587–596. 10.1016/j.neuron.2009.01.011 19249278 PMC2701743

[B76] Yamamoto J, Tonegawa S (2017) Direct medial entorhinal cortex input to hippocampal CA1 is crucial for extended quiet awake replay. Neuron 96:217–227.e4. 10.1016/J.NEURON.2017.09.017 28957670 PMC5672552

[B77] Zhang K, Ginzburg I, McNaughton BL, Sejnowski TJ (1998) Interpreting neuronal population activity by reconstruction: unified framework with application to hippocampal place cells. J Neurophysiol 79:1017–1044. 10.1152/jn.1998.79.2.1017 9463459

[B78] Zhang J, Hou L, Klann E, Nelson DL (2009) Altered hippocampal synaptic plasticity in the Fmr1 gene family knockout mouse models. J Neurophysiol 101:2572–2580. 10.1152/jn.90558.2008 19244359 PMC2681424

[B79] Zheng C, Bieri KW, Hsiao YT, Colgin LL (2016) Spatial sequence coding differs during slow and fast gamma rhythms in the hippocampus. Neuron 89:398–408. 10.1016/j.neuron.2015.12.005 26774162 PMC4731025

[B80] Zheng C, Hwaun E, Loza CA, Colgin LL (2021) Hippocampal place cell sequences differ during correct and error trials in a spatial memory task. Nat Commun 12:3373. 10.1038/s41467-021-23765-x 34099727 PMC8185092

[B81] Zielinski MC, Tang W, Jadhav SP (2020) The role of replay and theta sequences in mediating hippocampal-prefrontal interactions for memory and cognition. Hippocampus 30:60–72. 10.1002/hipo.22821 29251801 PMC6005707

